# Aspirin and colorectal cancer.

**DOI:** 10.1038/bjc.1997.444

**Published:** 1997

**Authors:** C. La Vecchia, E. Negri, S. Franceschi, E. Conti, M. Montella, A. Giacosa, A. Falcini, A. Decarli

**Affiliations:** Istituto di Ricerche Farmacologiche Mario Negri, Milan, Italy.

## Abstract

The relationship between aspirin use and colorectal cancer risk was examined by a case-control study in Italy. Regular aspirin use was reported by only 47 (3.5%) cases and 77 (4.1%) control subjects, giving a multivariate odds ratio (OR) of 0.7 (95% CI 0.5-1.0) after allowance for education, physical exercise and selected dietary factors.


British Journal of Cancer (1997) 76(5), 675–677
© 1997 Cancer Research Campaign

Short communication

### Aspirin and colorectal cancer

**C La Vecchia[1,2], E Negri[1], S Franceschi[3], E Conti[4], M Montella[5], A Giacosa[6], F Falcini[7] and A Decarli[2,8]**

[1]Istituto di Ricerche Farmacologiche 'Mario Negri', Via Eritrea 62, 20157 Milan, Italy; [2]Istituto di Biometria e Statistica Medica, Università degli Studi di Milano, Via Venezian 1, 20133 Milan, Italy; [3]Centro di Riferimento Oncologico, Via Pedemontana Occ. 12, 33081 Aviano (PN), Italy; [4]Servizio di Epidemiologia e Oncogenesi, Istituto Regina Elena per lo Studio e la Cura dei Tumori, Viale Regina Elena 291, 00161 Rome, Italy; [5]Servizio di Epidemiologia, Istituto Tumori 'Fondazione Pascale', Cappella dei Cangiani, 80131 Naples, Italy; [6]Istituto Nazionale per la Ricerca sul Cancro, Largo Rosanna Benzi 10, 16132 Genoa, Italy; [7]Divisione di Oncologia Medica, Ospedale Pierantoni, Via Forlanini 34, 47100 Forli, Italy; [8]Istituto Nazionale per lo Studio e la Cura dei Tumori, Via Venezian 1, 20133 Milano, Italy

**Summary** The relationship between aspirin use and colorectal cancer risk was examined by a case–control study in Italy. Regular aspirin use was reported by only 47 (3.5%) cases and 77 (4.1%) control subjects, giving a multivariate odds ratio (OR) of 0.7 (95% CI 0.5–1.0) after allowance for education, physical exercise and selected dietary factors.

**Keywords:** aspirin; non-steroidal anti-inflammatory drugs; colorectal neoplasms; pharmacoepidemiology

At least six case–control studies (Kune et al, 1988; Rosenberg et al, 1991; Suh et al, 1993; Muscat et al, 1994; Peleg et al, 1994), based on over 3700 cases, have suggested that the risk of colorectal cancer may be reduced in regular aspirin users. Likewise, four (Thun et al, 1991, 1993; Schreinemachers and Everson, 1994; Giovannucci et al, 1994, 1995) out of five (Paganini-Hill et al, 1989; Paganini-Hill, 1994) cohort studies showed a protection of between 20% and 40% among regular aspirin users, although in the American Nurses' Health Study (Giovannucci et al, 1995) the protection was only evident for frequent use (≥ four times per week) and after 20 years of use. Thus, the role of time factors, including latency, in the possible relationship between aspirin and colorectal cancer are not yet fully understood (Paganini-Hill, 1994; Greenberg and Baron, 1996). In addition, few studies have investigated the potential confounding role of factors such as diet and exercise. We have therefore examined aspirin use and certain lifestyle factors in a case–control study in Italy.

### SUBJECTS AND METHODS

The data were derived from a case–control study of colorectal cancer, conducted between January 1992 and June 1996 in six Italian areas: Greater Milan; the provinces of Pordenone and Gorizia; the urban area of Genoa; the province of Forli, in northern Italy; the province of Latina in central Italy; and the urban area of Naples, in southern Italy. Only 2.4% of cases and 3.2% of controls approached for interview refused to participate. Information on aspirin was included from January 1993.

Cases were 1357 subjects with incident, histologically confirmed colorectal cancer, admitted to the major teaching and general hospitals within the area of study. For 860 cases, the site of origin of the cancer was the colon and for 497 the rectum. Controls were 1891 subjects residing in the same geographical areas who had been admitted to the same hospitals in which cases had been identified for acute conditions unrelated to known or likely risk factors for colorectal cancer. Of these, 23% had traumatic conditions, 27% acute non-traumatic orthopaedic disorders, 19% acute surgical conditions, 22% eye diseases and 9% miscellaneous other illnesses, such as ear, nose and throat and dental disorders.

The structured questionnaire included information on personal characteristics, education and other socioeconomic factors, general lifestyle habits, such as smoking, alcohol and coffee consumption, a validated food frequency section (based on 79 foods, food groups or recipes; Franceschi et al, 1993), a few indicators of physical activity (occupational and leisure time), gynaecological and obstetric data, related medical history, and history of lifetime use of aspirin, including indication, time, frequency and duration of use before diagnosis of the disease which led to hospital admission. A comprehensive list of major aspirin-containing preparations (including most common combinations of multiple non-steroidal antiinflammatory drugs, NSAIDS) in Italy was supplied.

Odds ratios (ORs) of colorectal cancer, and the corresponding 95% confidence intervals (CI), according to various measures of aspirin use were derived using unconditional multiple logistic regression (Breslow and Day, 1980), including terms for study centre, sex, quinquennia of age, education (< 7, 7–11, ≥ 12 years), marital status (never married, married), family history of colorectal cancer (yes/no), measures of energy intake (quintiles), alcohol drinking (abstainer, plus tertiles), vegetable and meat intake (approximate tertiles) and an overall indicator of physical exercise (approximate tertiles).

### RESULTS

Table 1 gives the distribution of colorectal cancer cases and of the control group according to sex, age, education and family history. Cases were 793 men and 564 women, aged 23–74 years (median age 62 years); controls were 1253 men and 638 women, aged 20–74 years (median age 59 years). Cases reported a family history of colorectal cancer more frequently in first-degree relatives, while there was no appreciable difference for education.

*Received 6 December 1996*
*Revised 10 March 1997*
*Accepted 12 March 1997*

*Correspondence to:* C La Vecchia

**Table 1** Distribution of 1357 cases of colorectal cancer and 1891 controls according to sex, age and selected covariates. Italy, 1993–96

| | Colorectal cancer cases | | Controls | |
|---|---|---|---|---|
| | No. | % | No. | % |
| Sex | | | | |
| Male | 793 | 58.4 | 1253 | 66.3 |
| Female | 564 | 41.6 | 638 | 33.7 |
| Age | | | | |
| < 45 | 99 | 7.3 | 267 | 14.1 |
| 45–54 | 220 | 16.2 | 420 | 22.2 |
| 55–64 | 476 | 35.1 | 600 | 32.7 |
| 65–74 | 562 | 41.4 | 604 | 31.9 |
| Education (years) | | | | |
| < 7 | 727 | 53.6 | 986 | 52.1 |
| 7–11 | 352 | 25.9 | 550 | 29.1 |
| ≥ 12 | 278 | 20.5 | 355 | 18.8 |
| Family history of colorectal cancer[a] | | | | |
| No | 1224 | 90.2 | 1810 | 95.7 |
| Yes | 133 | 9.8 | 81 | 4.3 |

[a]$P < 0.01$.

Various measures of aspirin use are examined in Table 2. A total of 47 (3.5%) cases vs 77 (4.1%) controls reported regular use (more than four times per week for > 6 months) of aspirin. The corresponding multivariate odds ratio was 0.7 (95% Cl 0.5–1.0). The risk tended to decrease with increasing duration of use (OR = 0.6 for use > 2 years) and was somewhat lower among current users (OR = 0.6). No material pattern of risk was observed with reference to time since first, last use or indication (pain vs antithrombosis). The protection was appreciably, though not significantly, stronger for rectal (OR = 0.4) than for colon cancer (OR = 0.9). The association was also somewhat stronger in women (OR = 0.5) and below age 60 years (OR = 0.6), although the interaction terms were not significant.

### DISCUSSION

This study provides further support for the hypothesis that aspirin may decrease the risk of colorectal cancer, which is of interest as it is based on a southern European population with specific patterns of aspirin use and prevalence of exposure to dietary and other possible correlates of colorectal cancer.

**Table 2** Relationship between various measures of aspirin use and colorectal cancer risk. Italy, 1993–96

| | Colorectal cancer cases | | Controls | | Odds ratio (95% CI)[a] |
|---|---|---|---|---|---|
| | No. | % | No. | % | |
| Non-users | 1310 | 96.5 | 1814 | 95.9 | 1[b] |
| Regular users | 47 | 3.5 | 77 | 4.1 | 0.7 (0.5–1.0) |
| Duration of use (years)[c] | | | | | |
| < 2 | 14 | 1.0 | 20 | 1.1 | 0.9 (0.5–1.7) |
| ≥ 2 | 32 | 2.4 | 56 | 3.0 | 0.6 (0.4–1.0) |
| Time since first use (years)[c] | | | | | |
| < 5 | 19 | 1.4 | 29 | 1.5 | 0.8 (0.4–1.5) |
| ≥ 5 | 28 | 2.1 | 46 | 2.4 | 0.7 (0.4–1.2) |
| Time since last use[c] | | | | | |
| < 1 year and current | 25 | 1.8 | 47 | 2.5 | 0.6 (0.4–1.0) |
| ≥ 1 years | 21 | 1.5 | 28 | 1.5 | 0.9 (0.5–1.6) |
| Indication[c] | | | | | |
| Pain | 20 | 1.5 | 33 | 1.7 | 0.7 (0.4–1.2) |
| Antithrombosis | 25 | 1.8 | 43 | 2.3 | 0.7 (0.4–1.2) |
| Site | | | | | |
| Colon | 37 | 4.3 | – | – | 0.9 (0.6–1.4) |
| Rectum | 10 | 2.1 | – | – | 0.4 (0.2–0.9) |

[a]Obtained from multiple logistic regression equations including terms for age, sex, centre, education, body mass index, total energy, alcohol and meat intake, and physical exercise. [b]Reference category. [c]The sum of the strata does not add up to the total because of missing values.

© Cancer Research Campaign 1997

Although this is one of the largest case–control investigations of colorectal cancer and aspirin, the number of regular aspirin users (and hence the statistical power) is relatively low, reflecting the pattern of aspirin use in Italy. Some of the diagnostic categories of the controls may have been associated with increased NSAID use, however separate comparison of cases with each of the major diagnostic categories of controls yielded similar results, thus providing reassurance against potential selection bias. In particular, the frequency of regular aspirin users was 3.7% among subjects with traumatic conditions and 4.2% among those with non-traumatic orthopaedic diseases.

Among the strengths of the study are the similar catchment areas of cases and controls and the almost complete participation. Furthermore, the choice of hospital controls has the advantage with regard to the reliability and validity of drug use information, as cases and controls should be similarly sensitized towards various aspects of their past medical history (Paganini-Hill and Ross, 1982; Kelly et al, 1990). Finally, allowance for potential confounding factors, including measures of social class, an indicator of physical exercise and selected dietary factors did not materially modify any of the estimates.

Colorectal cancer is the commonest neoplasm in non-smokers of both sexes in western countries (Levi et al, 1994), and a 30% reduction of risk would be of considerable public health benefit. Plausible biological mechanisms exist for a protective effect of NSAIDS in colorectal carcinogenesis (Gann et al, 1993; Greenberg and Baron, 1993). However, although we tried to avoid or minimize the limitations of case–control studies, namely possible selection, information bias and confounding, a 30% reduction in an observational study of this kind is only modest, and long-term clinical trials may still be required to properly address the issue of aspirin and colorectal cancer.

### ACKNOWLEDGEMENTS

This work was conducted within the framework of the CNR (Italian National Research Council) Applied Project 'Clinical Applications of Oncological Research' (contracts nos. 96.00759.PF39, 96.00548. PF39 and 96.00701.PF39) and with the contributions of the Italian Association for Cancer Research and the Europe against Cancer Programme of the Commission of the European Communities. The authors thank Ms Judy Baggott, Ms M Paola Bonifacino and GA Pfeiffer Memorial Library staff for editorial assistance.

### REFERENCES

Breslow EN and Day NE (1980) *Statistical Methods in Cancer Research, Vol. 1. The Analysis of Case–Control Studies.* IARC Sci Publ **32**.

Franceschi S, Negri S, Salvini S, Decarli A, Ferraroni M, Filiberti R, Giacosa A, Talamini R, Nanni O, Panarello G and La Vecchia C (1993) Reproducibility of an Italian food frequency questionnaire for cancer studies: results for specific food items. *Eur J Cancer* **29A**: 2298–2305

Gann PH, Manson JE, Glynn RJ, Buring JE and Hennekens CH (1993) Low-dose aspirin and incidence of colorectal tumors in a randomized trial. *J Natl Cancer Inst* **85**: 1220–1224

Giovannucci E, Rimm EB, Stampfer MJ, Colditz GA, Ascherio A and Willett WC (1994) Aspirin use and the risk for colorectal cancer and adenoma in male health professionals. *Ann Int Med* **121**: 241–246

Giovannucci E, Egan KM, Hunter DJ, Stampfer MJ, Colditz GA, Willett WC and Speizer FE (1995) Aspirin and the risk of colorectal cancer in women. *N Engl J Med* **333**: 609–614

Greenberg ER and Baron JA (1996) Aspirin and other nonsteroid anti-inflammatory drugs as cancer-preventive agents. In *Principles of Chemoprevention*, IARC Conference, Lyon, November 6–10, 1995. *IARC Sci Publ* **139**: 91–98

Kelly JP, Rosenberg L, Kaufman DW and Shapiro S (1990) Reliability of personal interview data in a hospital-based case-control study. *Am J Epidemiol* **131**: 79–90

Kune GA, Kune S and Watson LF (1988) Colorectal cancer risk, chronic illnesses, operations, and medications: case control results from the Melbourne Colorectal Cancer Study. *Cancer Res* **48**: 4399–4404

Levi F, Lucchini F and La Vecchia C (1994) Worldwide patterns of cancer mortality, 1985–89. *Eur J Cancer Prev* **3**: 109–143

Muscat JE, Stellman SD and Wynder EL (1994) Nonsteroidal antiinflammatory drugs and colorectal cancer. *Cancer* **74**: 1847–1854

Paganini-Hill A (1994) Aspirin and the prevention of colorectal cancer: a review of the evidence. *Semin Surg Oncol* **10**: 158–164

Paganini-Hill A and Ross RK (1982) Reliability of recall of drug usage and other health-related information. *Am J Epidemiol* **116**: 114–122

Paganini-Hill A, Chao A, Ross RK and Henderson BE (1989) Aspirin use and chronic diseases: a cohort study of the elderly. *Br Med J* **299**: 1247–1250

Peleg II, Maibach HT, Brown SH and Wilcox CM (1994) Aspirin and nonsteroidal anti-inflammatory drug use and the risk of subsequent colorectal cancer. *Arch Inter Med* **154**: 394–399

Rosenberg L, Palmer JR, Zauer AG, Warshauer ME, Stolley PD and Shapiro S (1991) A hypothesis: nonsteroidal anti-inflammatory drugs reduce the incidence of large-bowel cancer. *J Natl Cancer Inst* **83**: 355–358

Schreinemachers DM and Everson RB (1994) Aspirin use and lung, colon, and breast cancer incidence in a prospective study. *Epidemiology* **5**: 138–146

Suh O, Mettlin C and Petrelli NJ (1993) Aspirin use, cancer, and polyps of the large bowel. *Cancer* **72**: 1171–1177

Thun MJ, Namboodiri MM and Heath CW Jr (1991) Aspirin use and reduced risk of fatal colon cancer. *N Engl J Med* **325**: 1593–1596

Thun MJ, Namboodiri MM, Calle EE, Flanders WD and Heath CW Jr (1993) Aspirin use and risk of fatal cancer. *Cancer Res* **53**: 1322–1327

© Cancer Research Campaign 1997


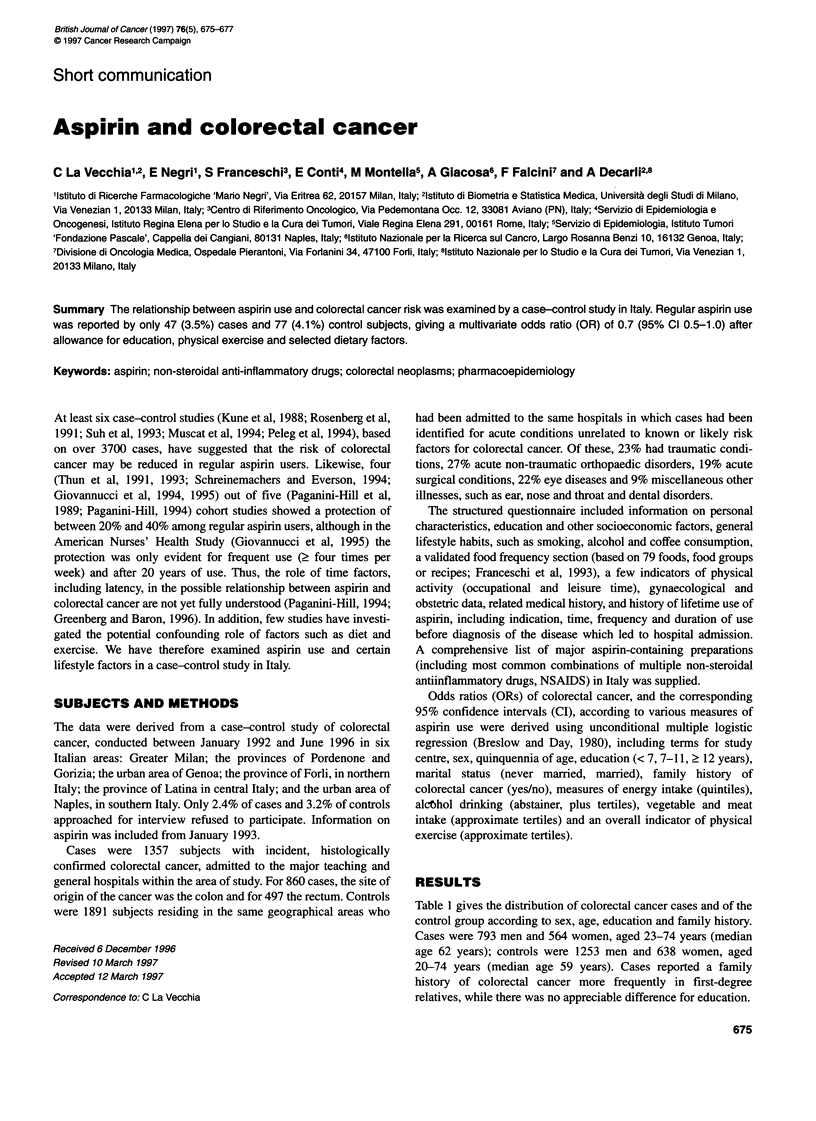

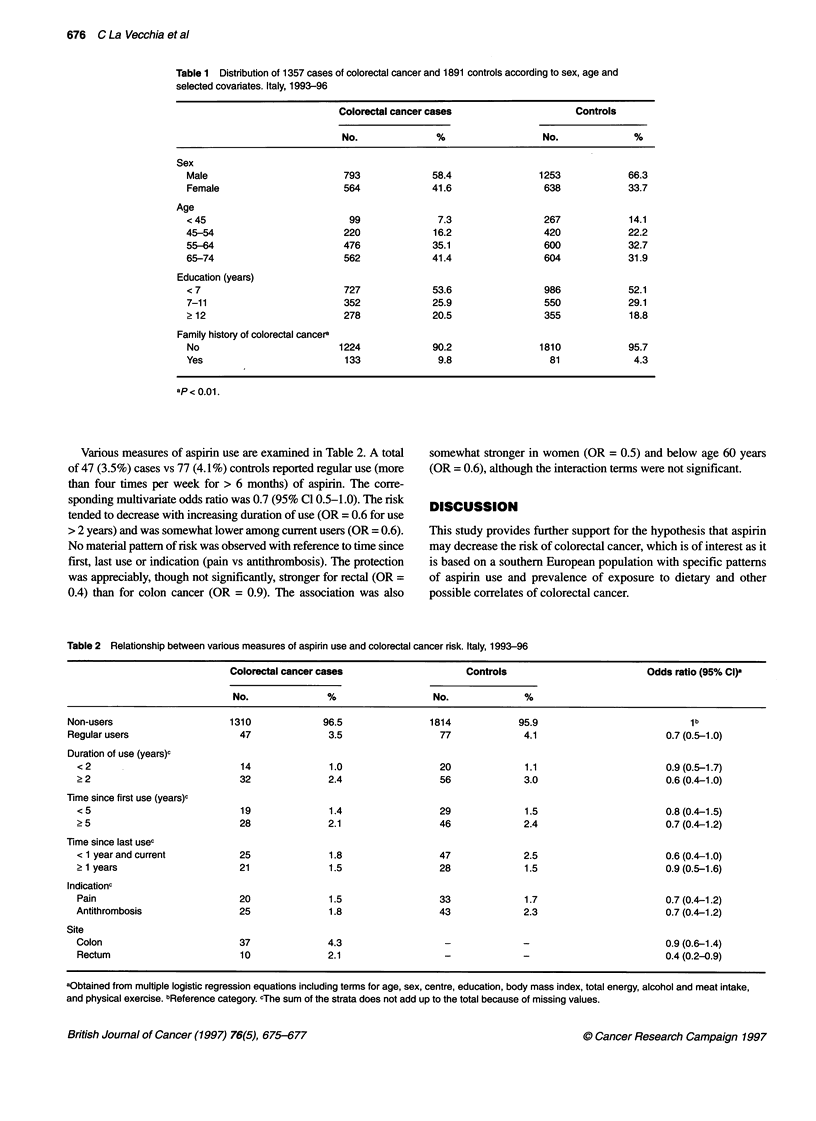

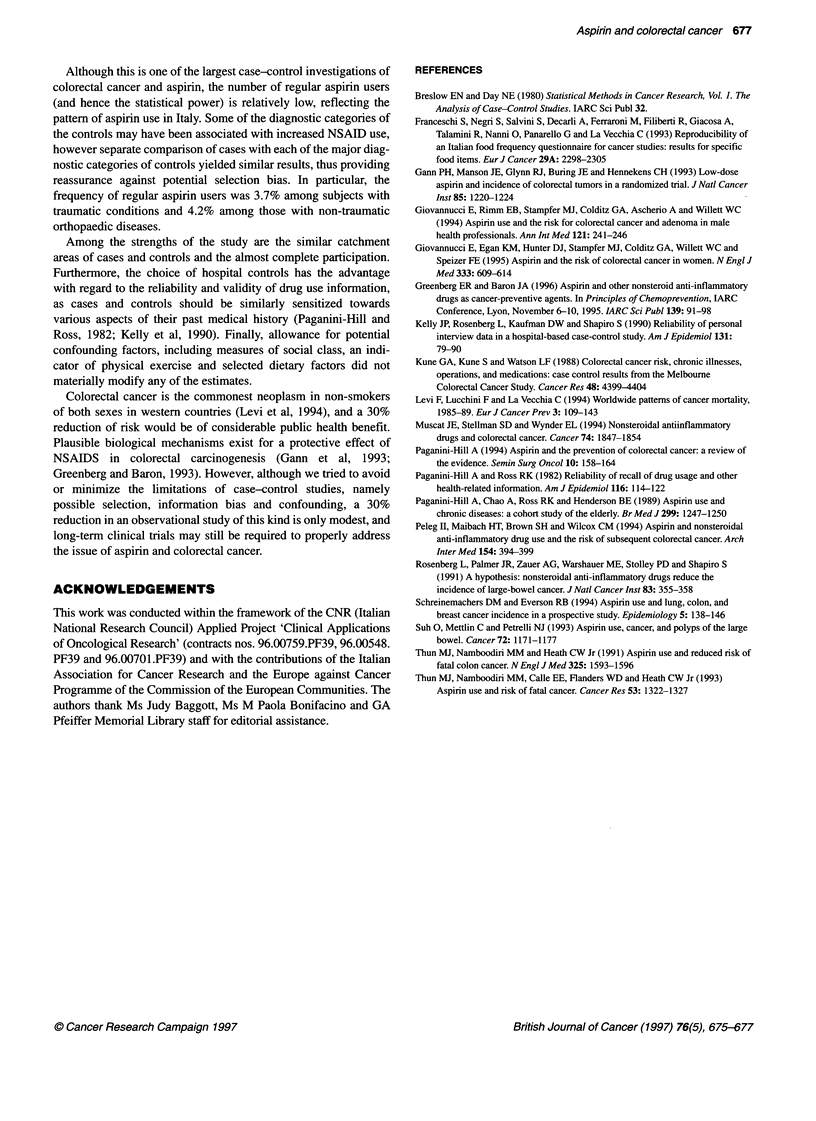


## References

[OCR_00325] Franceschi S., Negri E., Salvini S., Decarli A., Ferraroni M., Filiberti R., Giacosa A., Talamini R., Nanni O., Panarello G. (1993). Reproducibility of an Italian food frequency questionnaire for cancer studies: results for specific food items.. Eur J Cancer.

[OCR_00331] Gann P. H., Manson J. E., Glynn R. J., Buring J. E., Hennekens C. H. (1993). Low-dose aspirin and incidence of colorectal tumors in a randomized trial.. J Natl Cancer Inst.

[OCR_00341] Giovannucci E., Egan K. M., Hunter D. J., Stampfer M. J., Colditz G. A., Willett W. C., Speizer F. E. (1995). Aspirin and the risk of colorectal cancer in women.. N Engl J Med.

[OCR_00336] Giovannucci E., Rimm E. B., Stampfer M. J., Colditz G. A., Ascherio A., Willett W. C. (1994). Aspirin use and the risk for colorectal cancer and adenoma in male health professionals.. Ann Intern Med.

[OCR_00346] Greenberg E. R., Baron J. A. (1996). Aspirin and other nonsteroid anti-inflammatory drugs as cancer-preventive agents.. IARC Sci Publ.

[OCR_00351] Kelly J. P., Rosenberg L., Kaufman D. W., Shapiro S. (1990). Reliability of personal interview data in a hospital-based case-control study.. Am J Epidemiol.

[OCR_00356] Kune G. A., Kune S., Watson L. F. (1988). Colorectal cancer risk, chronic illnesses, operations, and medications: case control results from the Melbourne Colorectal Cancer Study.. Cancer Res.

[OCR_00361] Levi F., Lucchini F., La Vecchia C. (1994). Worldwide patterns of cancer mortality, 1985-89.. Eur J Cancer Prev.

[OCR_00365] Muscat J. E., Stellman S. D., Wynder E. L. (1994). Nonsteroidal antiinflammatory drugs and colorectal cancer.. Cancer.

[OCR_00369] Paganini-Hill A. (1994). Aspirin and the prevention of colorectal cancer: a review of the evidence.. Semin Surg Oncol.

[OCR_00377] Paganini-Hill A., Chao A., Ross R. K., Henderson B. E. (1989). Aspirin use and chronic diseases: a cohort study of the elderly.. BMJ.

[OCR_00373] Paganini-Hill A., Ross R. K. (1982). Reliability of recall of drug usage and other health-related information.. Am J Epidemiol.

[OCR_00381] Peleg I. I., Maibach H. T., Brown S. H., Wilcox C. M. (1994). Aspirin and nonsteroidal anti-inflammatory drug use and the risk of subsequent colorectal cancer.. Arch Intern Med.

[OCR_00386] Rosenberg L., Palmer J. R., Zauber A. G., Warshauer M. E., Stolley P. D., Shapiro S. (1991). A hypothesis: nonsteroidal anti-inflammatory drugs reduce the incidence of large-bowel cancer.. J Natl Cancer Inst.

[OCR_00391] Schreinemachers D. M., Everson R. B. (1994). Aspirin use and lung, colon, and breast cancer incidence in a prospective study.. Epidemiology.

[OCR_00395] Suh O., Mettlin C., Petrelli N. J. (1993). Aspirin use, cancer, and polyps of the large bowel.. Cancer.

[OCR_00403] Thun M. J., Namboodiri M. M., Calle E. E., Flanders W. D., Heath C. W. (1993). Aspirin use and risk of fatal cancer.. Cancer Res.

[OCR_00399] Thun M. J., Namboodiri M. M., Heath C. W. (1991). Aspirin use and reduced risk of fatal colon cancer.. N Engl J Med.

